# Professor W. G. Redman

**Published:** 1869-10

**Authors:** 


*Professor W, G. Redman.*—We are gratified to learn that the *Louisville Medical College* has added to its regular course of instruction, Lectures on Pharmacy, Medical Jurisprudence, and Elements of Dentistry, and that the latter chair has been offered to our friend W. G. Redman, D. D. S., of Louisville.

No better appointment could have been made, and we congratulate the faculty of this medical college upon their choice, Dr. Redman having accepted the position. Dr Redman's name is familiar to the dental profession as the inventor of Redman's Automatic Mallet.

